# Factors Influencing Parents' Preferences and Parents' Perceptions of Child Preferences of Picturebooks

**DOI:** 10.3389/fpsyg.2017.01448

**Published:** 2017-09-01

**Authors:** Laura Wagner

**Affiliations:** Department of Psychology, The Ohio State University Columbus, OH, United States

**Keywords:** picturebooks, preferences, content analysis, gender, parent-child reading

## Abstract

This study examined factors influencing parents' preferences and their perceptions of their children's preferences for picturebooks. First, a content analysis was conducted on a set of picturebooks (*N* = 87) drawn from the sample described in Wagner (2013); Then, parents (*N* = 149) rated the books and several content properties were examined for their ability to predict parents' preferences and their perception of their children's preferences. The initial content analysis found correlated clusters of disparate measures of complexity (linguistic, cognitive, narrative) and identified a distinctive sub-genre of modern books featuring female protagonists. The experimental preference analysis found that parents' own preferences were most influenced by the books' age and status; parents' perceptions of their children's preferences were influenced by gender, with parents perceiving their sons (but not daughters) as dis-preferring books with female protagnoists. In addition, influences of the child's reading ability and the linguistic complexity of the book on preferences suggested a sensitivity to the cultural practice of joint book-reading.

## Introduction

Picturebooks are an integral part of modern childhood in highly literate societies, and researchers have become increasingly interested in how these books work, how they are used, and how they influence social and developmental outcomes (see Horst and Houston-Price, 2015 and other papers within that special issue). The current work considers an aspect of picturebooks that has received less scholarly attention, specifically, what determines which picturebooks people *like*? It draws on the coded sample of books described in Wagner (2013) and asks if there are characteristic features of the books that parents like and/or believe that their children like.

There is no issue about whether people like picturebooks in general; their sales figures are consistently solid (Springen, 2010) and children's materials account for over 35% of library circulation nationwide (Institute of Museum Library Services, 2014). But picturebooks form a highly heterogeneous class that represent a range of styles and functions, and are beloved to different degrees. Moreover, they serve a heterogeneous audience consisting of both children as well as their parents (Bullen and Nichols, 2011). Relatively little work has been done examining parent and child preferences and whether those preferences can be systematically linked back to properties of the books, such as their narrative style, linguistic complexity, the gender of the characters, or the external validation the books have received.

One class of previous work has focused on the question of what books children themselves like. Some studies have found that children's preferences depend in part on relatively superficial properties, such as the presence of colored illustrations and bright colors (Malter, 1948; Rudisill, 1952; Brookshire et al., 2002) and children's choice of books may also depend on accidental properties, such as whether they appear at eye-level on a shelf (Reutzel and Gali, 1997). Considering more content-based aspects of books, educators and librarians have developed a rich set of expectations about what factors drive children's preferences (Langerman, 1990; Chall et al., 1996), but there are only a few genuine experimental investigations of what books children prefer—particularly for pre-school aged children. Robinson et al. (1997) let kindergarten and first grade children from low and middle-class SES backgrounds repeatedly select books to take home over the course of 2 months. The books were drawn from a specified set of 40 books encompassing multiple genres of books, including realistic and fantasy narratives, alphabet books, and non-fiction books; moreover, the books also varied in terms of difficulty level and popularity. Robinson et al. (1997) then analyzed what features drove children's choices. They found that children preferred books they were more familiar with, and also found that certain types of books were more favored; specifically, narrative fiction books with some fantasy elements were most popular. A more limited book-selection study was conducted by Mohr (2006) using just nine books that were selected to include both narrative and non-fiction books, as well as multi-cultural books that reflected the ethnicity of the children being tested. She asked first grade children to choose a single book from the set and found that they preferred non-fiction books about animals but no preferences based on the race/cultural ethnicity of the main characters of the books were found. However, as 46% of her children actually chose the same book (a non-fiction animal book, S. Simon's *Animals Nobody Loves*) her results may reflect the outsized popularity of a single item. Both Robinson et al. (1997) and Mohr (2006) noted some mild tendencies for girls and boys to favor different types of books, but neither found a significant gender effect in preferences.

The current study, however, does not examine directly what children prefer, but instead asks what books adults (parents, in particular) prefer and believe their children prefer. Millard (1997) documented how children's later enthusiasm and motivation to read is grounded in their early reading experiences with their parents (especially with their mothers), and it is reasonable to assume that parents' own preferences, as well as the preferences they perceive in their children, will influence those experiences. Moreover, picturebooks serve not only to enculturate children into the process of reading, but they also provide a window onto more general cultural processes (e.g., how to go to school, how to be a good friend) as well as cultural biases (e.g., appropriate behaviors for boys vs. girls). Parents' opinions about picturebooks matter because parents are critical curators of children's reading materials and it is important that we understand preferences from the parents' point of view.

Only one experimental investigation has directly examined parents' choices of picturebooks for their children. Anderson et al. (2001) asked 24 middle-class parents to choose from among a pre-selected set of 14 books what they would like to read to their 4-year-old child and explain their choices. The book set was quite diverse and included a range of genres, including fairy tales, other narratives, poetry, non-fiction, alphabet books, etc. The results suggested that prior familiarity with a book was a critical influence on parents' choices (the most frequently chosen book was the best known) and parents selected more informational (as opposed to narrative) books for boys than girls. Parents appealed explicitly to the topic/content of the book as being the most important factor, followed by their expectation of their child's interests and then the aesthetics of the book.

The results of Anderson et al. (2001) are intriguing, but the small size of the study is a clear limitation. The current work focuses on adding breadth to the question, by investigating more books than any previous study, more factors within those books, and querying more parents about those books. Experiment 1 reports the results of a content analysis of a large set (*N* = 87) of books to see how a range of content features that have been previously identified in the literature connect to each other across and between books. Experiment 2 asked a large set of parents (*N* = 149) to rate the books and explored the extent to which their ratings were accounted for by the content features of the books.

## Experiment 1: content analysis of picturebooks

The purpose of this experiment was two-fold. First, it explored how six factors are linked together within a large sample of actual picturebooks. As will be seen, in some respects these links correspond well to established book genres but in other ways, they suggest some unexpected connections. Second, it provided a data-driven characterization of what the parents in Experiment 2 were actually making judgments about. The results were thus critical for interpreting the results of the next experiment.

What kinds of factors might influence parents' preferences? The current work considered six factors that cover a range of picturebook features. These features were chosen because previous work has suggested that they influence the preferences of either children or adults, and in many cases, they have also been suggested as relevant criteria to teachers and clinicians as means of choosing books (Chall et al., 1996; Schwarz et al., 2015).

The first factor was Cultural Prominence. Books are cultural objects and they have varying degrees of overall popularity within our society, as measured by sales over time, awards earned, and spin-off products. There is some evidence that older children's book choices are influenced by peer suggestions and cultural currency (Moss and McDonald, 2004; Williams, 2008) and parents may similarly prefer some books simply because they are more culturally notable than others.

The second factor was the extent to which the book is a REFLECTION OF THE CHILD'S Experience. Parents may also prefer books that center on the child's life, or they may believe that children will prefer such books, regardless of what they themselves enjoy. This factor may also reflect a dimension of cognitive complexity, as children may have more difficulty with books that are more distal from their own experience (Schwarz et al., 2015).

The third factor was Character Gender. A variety of studies have considered how children might be influenced by gendered portrayals in books (Jennings, 1975; Ashton, 1983; see Abad and Pruden, 2013 for a review) and the role of parents in enculturating children into gender roles (Leaper, 2002; Tenenbaum and Leaper, 2002; Martin and Ruble, 2004; Kane, 2006). Examining how the gender of characters within preferred and non-preferred books may provide additional insight into these enculturation processes.

The fourth and fifth factors were related to complexity. Complexity was operationalized in two ways: Words per Page and Theory of Mind. The Words per Page measure provided a very rough estimate of linguistic complexity. While this measure is by no means the most sophisticated way to assess complexity, it provides a reasonable (and easy to code) heuristic: the denser the text is within the book, the more likely it is to contain a variety of word types and linguistic structures. The Theory of Mind measure provided an estimate for one important component of cognitive complexity. Children improve their ability to track mental states and reason about them throughout childhood and this skill is a hallmark of cognitive development (e.g., Wellman, 1990; Astington, 1993). Previous work (Cassidy et al., 1998) has found that picturebooks vary in the extent to which they require an understanding of the characters' mental states in order to be understood. The books were thus scored for the amount of mental state language and mentalistic concepts they contained. Parents may prefer more complex books to reflect their own superior understanding; alternatively, since even the most linguistically and mentalisitcally complex of the books were well within the capacities of the parents in our sample, they may show no influence of complexity at all. Regardless of their own preferences, parents may assume that better readers will prefer more complex books.

The final factor examined was Story Structure. This factor was partially inspired by the coding scheme in Nicoloupolou and Sawyer (in preparation) and similar ideas discussed in Schwarz et al. (2015). Picturebooks vary in terms of their story structure and indeed, whether they include a story at all. Some books contain a canonical problem-resolution structure (e.g., *Madeline*) while other books simply contain a sequence of temporally linked events with little that could be deemed a conventional story (e.g., *Maisy Goes to Preschool*) and still other books are wholly without a story of any kind (e.g., *I Love Colors*). Narratological investigations of picturebooks have largely focused on books with strong storylines (e.g., Nikolajeva and Scott, 2001; Wolfenbarger and Sipe, 2007) and these books are clearly preferred among many researchers. Parents might also be expected to prefer books with strong stories as these more closely reflect the types of books that adults read themselves. Parents' perceptions of their children's preferences, however, may diverge from their own.

### Books examined

The set of picturebooks that were judged by parents in Experiment 2 consisted of 87 books drawn from the set that is part of an extended program of content analysis (see Wagner, 2013). The books in the complete set were created as a curated set in 2011 with the intention of getting a wide variety of books that truly reflected children's reading experiences. Approximately half the books were drawn from suggestions made by parents in a survey at a local science museum; other books were selected to insure books were drawn from different time periods (see Table 1), covered different subject-matter (alphabet books, narrative books, etc.), had different levels of popularity as measured by Amazon.com rankings, and included not only books with highbrow validation (e.g., Caldecott winners) but also those with pop-culture validation (e.g., books with ties to movies and TV shows). In addition, no author was allowed more than one narrative and one non-narrative book in the set with the exception of Dr. Seuss whose work was so overwhelmingly popular that three of his narrative books were included. The overwhelming majority (93%) of the books were available for sale new on Amazon.com. The sample has a reasonable amount of overlap with the recently described Infant Bookreading Database (IBDb) (Hudson Hudson Kam and Matthewson, 2016) which was generated through surveys of parents. Of the 87 books in the current sample, 20 were among the 105 most frequently mentioned books in the IBDb and an additional 6 were near matches (e.g., Scarry's *Busytown: Cars and Trucks and Things that Go* was among the IBDb's most common books but the current sample contained Scarry's *The Busiest Firefighters Ever*).

**Table 1:** Summary of the sample of books used.

		**Classic 1900–1979**	**Intermediate 1980–2004**	**Contemporary 2005–2011**	**Totals**
Number of books (% of total)		24 (28%)	35 (40%)	28 (32%)	87 (100%)
**Acclaim**	No Awards	19 (79%)	16 (46%)	17 (61%)	52 (60%)
*N of books (% of column)*	Other Awards	3 (13%)	11 (31%)	7 (25%)	21 (24%)
	Caldecott	2 (8%)	8 (23%)	4 (14%)	14 (16%)
**Mean Reflection of the Child's Experience Score (sd)**		2.9 (0.7)	2.3 (0.5)	2.4 (0.6)	2.5 (.2)
*Scores range from 1 (furthest from child's experience) to 4 (closest to the child's experience)*					
**Gender of Main Character(s)**	Male Only	14 (58%)	10 (29%)	8 (29%)	32 (37%)
*N of books (% of column)*	Mixed/Unspecified	9 (38%)	19 (54%)	15 (53%)	43 (49%)
	Female Only	1 (4%)	6 (17%)	5 (18%)	12 (14%)
**Mean Number of Words per Page (sd)**		19.6 (19.1)	23.8 (24.3)	19.8 (19.9)	21.4 (21.4)
*Range is 1.03–114 words per page*					
**Mean Theory of Mind Score (sd)**		3.3 (0.8)	2.7 (1.1)	2.7 (1.0)	2.9 (0.97)
*Scores range from 1 (least theory of mind) to 4 (most theory of mind)*					
**Story Structure**	No Story	1 (4%)	2 (6%)	6 (21%)	9 (10%)
*N of books (% of column)*	Temporal Sequence	4 (16%)	12 (34%)	8 (29%)	24 (28%)
	Regular Story	16 (67%)	16 (46%)	11 (39%)	43 (49%)
	Traditional Story	3 (13%)	5 (14%)	3 (11%)	11 (13%)

*The break-down into Classic, Intermediate, and Contemporary age groups are presented to provide a holistic picture of secular changes across the books. All analyses involving the age of the book used the exact year of publication*.

### Coding procedures and reliability

All books were coded for each element by two independent coders. All points of disagreement were reviewed by a third coder and the code was resolved by majority rule. In the handful of cases where all three coders disagreed, the author made a final resolution. Where the codes depended in some part on subjective judgments (e.g., not the year the book was published), Kappa scores were calculated and are reported below.

### Coding categories and descriptions

#### Cultural prominence

This factor was broken down into 2 separate sub-categories: the Age of the book and the Acclaim the book generated from critics.

##### Year published

A book's age serves as a proxy for ongoing cultural importance: A book originally published in 1939 that is still available for sale or still recommended by parents suggests that book has some special cultural status. The original date of each book's publication was noted. These ranged from 1907 (*Tale of Tom Kitten*) to 2011 (*I Am Sheriff Woody*). Table 1 classifies these books into publication age groups: Classic Books (published between 1907 and 1979) included books that would likely have been available to the parents in our survey when they were children themselves; Intermediate Books (published between 1980 and 2004) are those falling between the other two age categories; and Contemporary Books (published between 2005 and 2011) included those which were published during the lifetime of the target children in the survey. For all statistical analyses, however, the actual year of publication was used.

##### Acclaim

This code marked the prestige of the awards bestowed on each book. The Caldecott books included both Caldecott winners and Caldecott honor books (*The House in the Night*); the Other Award code was used for books that received other kinds of awards (such as being an MLA Notable book) but had not made the Caldecott lists (*The Library Lion*); and the No Award code was used for books that had received no prestigious awards (*I Just Forgot*). For analysis purposes, these codes were assigned numerical values from 1 to 3, with higher numbers reflecting a greater amount of critical acclaim.

#### Reflection of child's experience

This score was based on the combination of five categories within the larger sample: The Species of the main character, the Age of the main character, the Physical setting of the story, the Temporal setting of the story, and the Overall Realism of the story. The composite score reflected how closely the main character's life and story were to the average child reader. The Very Near code (e.g., *No, David!)* indicated that the book was a close reflection of the child's life and required the main character to be a human child; the story was required to take place in locations a pre-school or grade-school aged child would be readily familiar with (such as a home, school, playground, or store); the story was also required to have a contemporary time setting as reflected in contemporary clothing and artifacts; and there could be no fantasy elements in the story. The Almost Near code was used when the book met all of the requirements for the strict Very Near code except that the main character was in fact a personified animal; for example, in *Olivia*, Olivia is actually a pig but in all other respects, the book passes the tests for being very near to the child's experience. The Intermediate code was used if the book failed 1 or 2 of the closeness criteria beyond starring a personified animal. For example, *Where the Wild Things Are* contains fantasy elements that break the sense of day-to-day realism and in addition, large portions taking place in a more exotic physical location. Finally, the Far code was used when the book failed 3 or more of the criteria; for example, *Shrek!* fails them all as it stars an adult, non-human, in a non-contemporary time period, in an exotic location, where magic is possible. The Cohen's Kappa statistic found “good” agreement between the coders, *k* = 0.73 (95% CI, 0.66 −0.79); 12 cases were not resolved by the 3rd coder and required additional review. For analysis purposes, these codes were assigned numerical values from 1 to 4, with higher numbers indicating increasingly close reflections of the child's experience.

#### Gender of main characters

The main character (or characters) were defined as the characters who were the primary focus of the action of the narrative and appeared on most of the pages of the book. The gender of these characters was determined through the use of pronoun reference and when necessary, gender stereotypic clothing in the pictures. When a clear determination of gender could not be made (*Chicka Chicka Boom Boom)* the characters were coded as being unspecified for gender. Books that had no clear protagonists—either because they had no characters at all (*Eating the Alphabet*) were also coded as being unspecified for gender. Books which had multiple protagonists of different genders (*The Cat in the Hat*) were coded as Mixed. For analysis purposes, gender was coded on an increasing numerical scale, with males coded as 1, the mixed and unspecified cases as 2, and the females as 3; thus larger numbers indicate increasing amounts of femaleness in the books.

#### Words per page

This code was used as a proxy for linguistic complexity, as noted above. It was calculated by dividing the total number of words by the total number of pages in the book. The score was estimated manually by the two coders and they were considered to agree if both coders' values were within 10% of the mean across the coders. The book with the fewest words per page (1.03) was *The Lion and the Mouse;* the book was the most (114) was *Thumbelina*.

#### Theory of mind

This score was based by combining two coding categories from the complete sample: the amount of mentalism needed to interpret the book and the narrative perspective taken within the book. Mentalism was broadly construed to include a full range of mental states, including thoughts, emotions, intentions, and perceptions. In general, mentalism was identified through the use of explicit mentalistic words in the text (e.g., “think,” “want,” “was sad”) but some situations (e.g., purposeful deception) would also be considered. Narrative perspective was deemed important because different kinds of narrators have different access to the characters' inner states. First-person and omniscient narrators are intrinsically more mental-state oriented because they explicitly provide the interior perspective of the main character (first-person) or multiple characters (omniscient); these narrators have access to characters' mental states and use them to explain actions. By contrast, an external narrator does not describe interior states, although they sometimes draw mentalistic inferences based on external properties (“she looked angry”). The Absent code was used for books that contained no mental agents—that is, no characters (people, animals, or even anthropomorphized objects) who were capable of thoughts, emotions, or even communicative abilities (e.g., *Airplanes: Soaring! Diving! Turning!)*. For these books, no understanding of mental states was required to follow the book's content. The Low code was used for books that did have mental agents present, but the narrator took an external perspective and there were no (or extremely few) explicit references to mental states (e.g., *Hippos Go Berserk)*. In these books, there were characters who presumably did have mental lives but the book's text only described the externally visible actions of the characters. Thus, it was possible to follow the action of these books without an understanding of mental states, although there were potential opportunities to impose a mentalistic interpretation on the events. The Intermediate code was used for books containing first-person or omniscient narrators but nevertheless contained very few or no explicit references to mental states as well as a handful of books using external narrators (who had no direct access to the characters' inner states) but that did include moderate levels of explicit mental state references (e.g., *1 Zany Zoo)*. To understand these books, it was necessary to have some understanding of mental states, but that understanding was not necessarily called upon throughout each book. Finally the High code was used for books containing many mental state references in the text and used first person or omniscient narrators who had direct access to the characters' mental states (e.g., *Harold and the Purple Crayon)*. These books would be extremely difficult to understand without a robust understanding of mental states and how they worked. The Cohen's Kappa statistic found “moderate” agreement between the coders, *k* = 0.51 (95% CI, 0.38–0.63); nine cases were not resolved by the 3rd coder and required additional review. For analysis purposes, these codes were assigned numerical values from 1 to 4, with higher numbers reflecting increasing amounts of theory of mind needed to interpret the content.

#### Story structure

This factor draws from the field of narratology and indicates the extent to which the book contains a well-formed narrative. At the bottom end of this category were books that contained No Story at all—this code was used for some alphabet books, but also for books that simply consisted of temporally unrelated facts (*Elmo Loves You* consists of a list of things Elmo and his friends love). The Temporal Sequence code was used for books that contained a temporal sequence of events but no kind of causal arc driven by the events within the story. For example, *Maisy Goes to Preschool* follows Maisy through a day of sequential events at pre-school. The events are ordered and related insomuch as they all happen over the course of a day, but for the majority of events, reversing their temporal order would not be fundamentally change the story's arc: there is no special causal reason that art class must precede recess as opposed to the other way around. The Regular Story code was used for books that contained an internally driven causal arc of any type but did not meet the very high narrative standards of the final category (*The Polar Express*). Many of these stories had canonical problem-resolution structures, but all that was required was for the order of the events to be causally meaningful (one must lose something before one can find it; one must buy a ticket before getting on the train, etc.). The top category of Traditional was reserved for books that not only contained a story-driven causal arc but also contained a clear moral, and conveyed the story through conventional linguistic means (i.e., not primarily as an interchange of dialogue between characters; *Ella the Elegant Elephant*). The Cohen's Kappa statistic found “moderate” agreement between the coders, *k* = 0.58 (95% CI, 0.45–0.70; seven cases were not resolved by the 3rd coder and required additional review^1^. For analysis purposes, these codes were assigned numerical values from 1 to 4, with higher numbers reflecting increasing amounts of story structure.

### Results and discussion

Table 1 summarizes the different factors across the books in the current sample. To determine the extent to which these factors were inter-related among each other, a Pearson's correlation was conducted using all of the factors. The full correlation matrix is shown in Table 2. As can be seen, there were several significant correlations among these factors.

**Table 2:** Pearson correlation analyses of the book content factors.

	**Year published**	**Acclaim**	**Child's experience**	**Gender of characters**	**Words per page**	**Theory of mind**
Year Published						
Acclaim	0.174					
Child's Experience	0.265^*^	0.008				
Gender of Characters	0.234^*^	0.006	0.266^*^			
Words Per Page	0.011	−0.028	−0.194	0.037		
Theory of Mind	−0.157	0.05	−0.192	−0.256^*^	0.409^***^	
Story Structure	−0.179	0.211	−0.425^***^	−0.147	0.346^***^	0.544^***^

Significance values are marked in the table (df = 85):

* p < 0.05;

^**^p < 0.01;

*** *p < 0.001. Unmarked values had p > 0.05. Please note, the variables were set up in general so that larger values indicated increasing amounts of the described quality—more theory of mind present, more traditional story, more of a reflection of the child's experience, more words per page, more acclaim etc.). In addition, gender was coded so that lower numbers indicated male characters and higher numbers indicated more female characters (thus, books published more recently are more likely to feature female characters and increased theory of mind is linked to more male characters)*.

First, Story Structure, Words per Page, and Theory of Mind were all strongly, positively correlated with each other. These mutually reinforcing connections suggest that there are important links among linguistic complexity, cognitive complexity and the telling of a traditional story. These relationships fit with narratological ideas that a classic story not only contains some kind causal arc, but also appeals to the mental states of its characters to explain the story's action. It also appears that it takes a certain amount of linguistic complexity to accomplish these goals. A further negative correlation between Story type and the Child's Experience points to the common sense notion that one of the things we expect from a traditional children's story is a little bit of magic and wonder. Thus, more traditional stories were less likely to closely reflect the child's own life. This clustering of features corresponds roughly to the features in Schwarz et al. (2015) that led speech language pathologists to classify a book as being comparatively “easy” (shorter sentences, less complex story structure, fewer abstract concepts, and greater similarity to the child's own experience) vs. “hard” (greater complexity in the story structure and language as well as more distance from the child's experience).

Second, there was set of correlations found involving the Gender of the Main Characters: this variable was negatively correlated to the amount of Theory of Mind in the book and positively correlated to the amount of the Child's Experience reflected in the book. As larger Gender values indicate more female characters, these links mean that male characters tend to be in books involving more theory of mind and female characters tend to be in books more directly reflecting the child's experience. These links suggest that there may be a coherent sub-genre within the picturebook set, consisting of books which describe the every-day lives of girls. A few example books in this sub-genre from the current sample are *Maisy Goes to Pre-school, Barney and Baby Bop Go to School*, and *My Best Friend is Belle*. This sub-genre of books is also correlated with the age of the book, and is most common among more recently published books. It is unclear why such a sub-genre might just be a modern one. The increase in books featuring female characters (or at least mixed-gender or a gender-unspecified casts of characters) in recent years could well be a sign of gender-equity progress in children's literature [but see Gooden and Gooden (2001) and Narahara (1998) for evidence that male characters dominate children's books, including award-winning ones]. The emphasis on everyday life may represent a secular trend toward realism in picturebooks, but it also seems possible that this type of book simply gets dated more quickly and therefore does not stand the test of time (or remain in active sales). More historically oriented work would be needed to see if similar books were commonly published in the past and simply went out of print.

Taken altogether, these results suggest that there may be identifiable genres of picturebooks with consistent clusters of properties. These clusters appear to depend both on cognitive constraints of the child (e.g., the complexity factors) as well as on larger cultural considerations (e.g., the gender correlations). The implications of these clusters will be taken up in the general discussion. Experiment 2, investigates whether these factors have any systematic influences on parents' preferences for books or on their perceptions of their children's preferences.

## Experiment 2: parents' preference judgments

This survey based study asked parents to assess each of the picturebooks and, for books they were familiar with, indicate how much they liked it and how much they thought their child liked it. These preference scores were then analyzed in relation to the properties within those books discussed in Experiment 1.

### Participants

This study was carried out with the approval of the Ohio State University Social and Behavioral Sciences IRB. Parents were recruited through social media postings and through a mailing to people who had previously brought their children to participate in research at an on-campus site. Participants were provided with written informed consent in accordance with the Declaration of Helsinki. They could opt into a lottery for a gift certificate to a children's bookstore as an incentive. A total of 178 individuals opened the survey but the data from 29 individuals (16%) were not included in the analysis because (1) they failed to provide even familiarity judgments for 90 of the books and thus left the majority of the survey completely blank (*N* = 14; 8%), (2) they provided familiarity judgments for at least 11 books but indicated that they were familiar five or fewer of the books (*N* = 4; 2%) and thus their scores had the potential to skew the sample, or (3) the age of their target child was over 11 years old (*N* = 11; 6%), and thus the parents' ratings very probably reflected retrospective judgments about their children rather than current ones^2^. The final sample consisted of 149 individuals.

### Methods

The judgments were collected through Survey Monkey. Following informed consent information, the first section of the survey asked participants to provide their own gender and the number of children they had. They were then asked to focus on a single child (the target child) for the remainder of the study—if they had more than one child, they were explicitly told to “pick one and think about that child for the whole study.” For the target child, participants were asked to provide the child's gender, age, reading level (on a scale from 1 [“My child does not yet know any letters”] to 5 [“My child can read a book on his/her own”]) and frequency of joint reading (on a scale from 1 [“I do not read with my child”] to 5[“I read at least one book with my child every day”]).

Following the initial section, participants saw one screen for each of 100 books from the set described in Wagner (2013) (and see Experiment 1 for more details). The order of the books was randomized for each participant. Each screen showed an image of the cover of the book; the title and author were also typed out on the screen. Participants first indicated if they were familiar with the book (“yes/no”). If participants were not familiar with the book, they were asked to skip to the next book in the survey. If participants were familiar with the book, they were asked to provide two preference ratings for it: “How much do YOU like this book” and “How much does YOUR CHILD like this book.” Both preference ratings used the same scale from 1 to 5 with labels provided for the scale's bottom (“I really do not like this book”), middle (“It's a pretty good book. I like it”), and top (“I love it! It's one of my favorites!”); for the question about the child's preferences, the phrasing was changed to the 3rd person (e.g., “He/She loves it!” “He/She really does not like this book”). In addition participants were asked to indicate how often they read the book with their child from 1 (“We never read this book”) to 5 (“We have read it many times”).

Participants were allowed to provide ratings for as many (or few) books as they chose to, although as noted above, participant who were not familiar with at least 5 books were not included in the final sample. Moreover, to help insure that participants were not making nostalgic judgments about children who were well past the age of reading picturebooks, participants were also eliminated if they indicated their target child for the survey was over 11 years old.

### Results

#### Participant properties

The overwhelming majority of the participants (87%) were mothers and participants had an average of 1.9 children. The average age of the target children was 5.1 years, with a standard deviation of 2.5 years. Approximately half of the target children were girls (*N* = 80 or 54%) and there were no significant differences between boys and girls in terms of age [Girls = 5.3 years; Boys = 4.8 years; *F*_(1, 147)_ = 1.32, n.s.], reading ability [Girls = 3.5 out of 5; Boys = 3.3 out of 5; *F*_(1, 147)_ = 0.83, n.s.], or frequency of joint reading [Girls = 4.4 out of 5; Boys = 4.6 out of 5; *F*_(1, 147)_ = 1.7, n.s.]. On average, the target children were thus pre-school aged, jointly read with their parents daily, and parents judged them able to do basic decoding but unable to read on their own.

The frequency of joint reading category proved to be somewhat problematic, however, as it was negatively correlated with age (Pearson *r* = −0.47, *n* = 147, *p* < 0.001) and reading ability (Pearson *r* = −0.43, *n* = 147, *p* < 0.001). Inspection of the data revealed a roughly step-wise function, with the mean frequency of joint reading for children under the age of 7 years being extremely high (4.8 out of a maximum score of 5) compared to simply being very high for the older children (*M* = 3.8). In retrospect, this makes sense: older children who can read on their own are less likely to participate in joint parent-child reading sessions. However, as the survey did not ask for any clarification about the nature of the joint parent-child reading, it is difficult to know precisely how to interpret this connection; more generally, this result calls into the question the validity of the reading frequency question asked for the individual books: joint reading frequencies at best would signal preference information in complex interactions with the child's age and ability. We therefore opted to omit it from further analyses and focused on the direct measures of preferences obtained.

#### Familiarity

The first analysis involving the books asked how familiar participants were with the sample chosen. For 13 of the books, five or fewer participants were familiar with the book and this was deemed too small a number to provide reliable preference information and further risked the possibility that these books would inappropriately skew the results. These 13 books were therefore removed from the set and all analyses—both in the content analysis described in Experiment 1 as well as in the data reported below—were based on the 87 remaining titles^3^. Of the final set of 87 books, each participant was familiar with an average of 32.9 books (range: 6–74) and each book was familiar to an average of 56.2 participants (range: 6–139).

#### General preferences

The books that received the highest and lowest preference scores for both adults and their children are shown in Table 3. As can be seen, there was some overlap across the parents' own preferences and children's perceived preferences, and in fact, there was a significant positive correlation between parent and child preference ratings over the full set of books (Pearson *r* = 0.403, *n* = 87, *p* < 0.001). However, this correlation was far from perfect, demonstrating that parents can and do separate what they like themselves and what they believe their children like.

**Table 3:** Most and least preferred books.

	**Parents' own preferences**	**Children's perceived preferences**
Five most preferred books		
	• The Monster at the End of this Book (4.04)	• Airplanes: Soaring! Diving! Turning! (4.33)
	• The Very Hungry Caterpillar (4.01)	• JUMP! (4.33)
	• Click, Clack, Moo: Cows that Type (3.9)	• Goodnight Gorilla (4.23)
	• Green Eggs and Ham (3.89)	• Dino-Baseball (4.18)
	• Goodnight Gorilla (3.88)	• The Very Hungry Caterpillar (4.07)
Five least preferred books		
	• Skeleton Meets Mummy (2.6)	• The Hello Goodbye Window (2.85)
	• My Best Friend is Belle (2.38)	• Barney and Baby Bop Go to School (2.5)
	• Thomas the Tank Engine's ABC's (2.33)	• Thumbelina (2.48)
	• Happy Birthday, Dora! (2.25)	• Dumbo (2.46)
	• Barney and Baby Bop Go to School (2.18)	• Tale of Tom Kitten (2.45)

*The five books that parents most and least preferred and that parents perceived their children as most and least preferring. Mean preference scores are in parentheses; scores could range from a low of 1 to a high of 5. Full author and publication information for all picturebooks mentioned in this paper can be found in the Appendix*.

#### Preference analysis

A linear mixed model was conducted with two fixed factors coming from features of the participants (the Target Child's Gender and the Target Child's Reading Level), five fixed factors coming from features of the books (Acclaim, Reflection of the Child's Experience, Gender of the Main Characters, Theory of Mind, and Story Structure), and two co-variate factors for the two continuous measures of Year of Publication and Words Per Page. Subjects and books were entered as random effects. The nature of the data collection and the large number of factors made a fully factorial analysis impossible, so the model contained only the main effects for the factors and a select set of interaction terms. Two interactions were predicted to arise from the interplay between properties of children and properties of the books, specifically in the domain of gender (Target Child's Gender by Gender of the Main Characters) and linguistic ability (Target Child's Reading Level by Words Per Page). Moreover, based on the previous correlational analysis, the interactions of Target Child's Gender by Reflection of Child's Experience and by Theory of Mind were also included because the latter two factors correlated in different directions with the Gender of the Main Characters and thus may have differential influences based on the gender of the target child. Separate models were created for the parents' own preferences and for the perceived preferences of their children. Tables 4, 5 show the omnibus values and significance levels for the two models; for every factor that was significant, the effect estimates for the levels (along with the reference level used) are also provided.

**Table 4:** Results for parents' preferences.

**Factor**	**df**	***F*-value**	**Significance**
Target Child's Gender	252.32	1.11	*p > 0*.10
Target Child's Reading Level^*^ Reference level is 3 – the intermediate reading level * Level 1: b = 0.38, t = 2.6, p < 0.009* * Level 2: b = 0.10, t = 0.82, p > 0.10* * Level 4: b = −0.06, t = −0.41, p > 0.10* * Level 5: b = 0.28, t = 2.7, p < 0.008*	201.63	3.64	*p* = 0.007
Acclaim^*^ Reference level is 1 – No Awards Given * Level 2 (some awards): b = 0.29, t = 2.6 p < 0.01* * Level 3 (Caldecott-level award): b = 0.44, t = 3.5, p < 0.001*.	66.33	7.48	*p* = 0.01
Reflection of the Child's Experience	67.91	0.19	*p > 0*.10
Gender of the Main Characters^*^ Reference level is 1 – Male Protagonist * .Level 2 (Mixed/Unspecified): b = 09, t = 0.82, p > 0.10* * Level 3 (Female): b = −0.27, t = −1.6, p > 0.10*	69.76	3.96	*p* = 0.023
Theory of Mind	68.78	1.44	*p > 0*.10
Story Structure	70.62	0.53	*p > 0*.10
Year Published^*^ b = −0.005, t = −2.4, p < 0.021	71.72	5.61	*p* = 0.021
Words per Page	78.80	1.11	*p > 0*.10
Reading Level × Words per Page^*^	4,665.94	7.06	*p < 0*.001
Target Child's Gender × Gender of Main Characters	4,659.87	1.22	*p > 0*.10
Target Child's Gender × Reflection of Child's Experience	4,647.30	0.51	*p > 0*.10
Target Child's Gender × Theory of Mind	4,656.47	0.70	*p > 0*.10

*All fixed factors and interactions with significant effects are marked with an asterisk. For each omnibus effect that was significant, the effect estimate value and significance is provided; note that these values and valences should be interpreted relative to the reference level indicated. For the interpretation of significant interactions, see text*.

**Table 5:** Results for children's perceived preferences.

**Factor**	**df**	***F*-value**	**Significance**
Target Child's Gender^*^ Reference Level is Female * Level 1 (Male): b = −1.1, t = −8.7, p < 0.001*	246.26	7.02	*p* = 0.009
Target Child's Reading Level	201.79	1.77	*p* > 0.10
Acclaim	70.34	0.76	*p* > 0.10
Reflection of the Child's Experience	72.44	0.37	*p* > 0.10
Gender of the Main Characters^*^ Reference level is 1 – Male Protagonist * Level 2 (Mixed/Unspecified): b = −0.08, t = −0.79, p > 0.10* * Level 3 (Female): b = −0.03, t = −0.20, p > 0.10*	73.57	5.9	*p* = 0.004
Theory of Mind	71.74	0.3	*p* > 0.10
Story Structure	74.67	2.25	*p* = 0.089
Year Published	78.24	0.73	*p* > 0.10
Words per Page^*^ b = −0.01, t = −4.3 p < 0.001	99.43	14.42	*p* < 0.001
Reading Level × Words per Page^*^	4,331.3	5.02	*p* < 0.001
Target Child's Gender × Gender of Main Characters^*^	4,294.84	35.02	*p* < 0.001
Target Child's Gender × Reflection of Child's Experience^*^	4,284.24	3.36	*p* = 0.018
Target Child's Gender × Theory of Mind	4,291.5	1.69	*p > 0*.10

*All fixed factors and interactions with significant effects are marked with an asterisk. For each omnibus main effect that was significant, the effect estimate value and significance is provided; note that these values and valences should be interpreted relative to the reference level indicated. For the interpretation of significant interactions, see text*.

#### Parents' preferences

As can be seen in Table 4, parents' own preferences were influenced by the cultural prominence of the book, as measured by the level of critical Acclaim and the year in which the book was published. There was a significant negative correlation between parents' ratings and the age of the book (Pearson's *r* = –0.28, *n* = 87, *p* < 0.011) reflecting the fact that parents preferred older books: as the year of publication got bigger (and thus the book was newer), parents' preference rating went down. For the acclaim variable, separate Tukey's *post-hoc* tests over books found that books that had won no awards at all were significantly dis-preferred (Mean preference rating = 3.2) relative to books that had books on the Caldecott list (*M* = 3.6; *p* < 0.007) but there was no significant difference between books that had won some awards (*M* = 3.4) and either of the other acclaim categories.

The only property of the books' contents that influenced parents' preferences was the gender of the main character, but the individual levels only differed from each other in *post-hoc* tests when they were analyzed independently from the other factors. Thus, an ANOVA of parents' preferences with the main character's gender as an independent variable was significant [*F*_(2, 84)_ = 4.4, *p* < 0.015] and Tukey's *post-hoc* tests over books found that parents dis-preferred books starring female protagonists (*M* = 2.9) relative to gender neutral books (*M* = 3.3; *p* < 0.031) and relative to books starring male protagonists (*M* = 3.4; *p* < 0.012) but were indifferent between the latter two types of books.

The remaining factors that influenced parents preferences were the target child's reading ability, although this influence was complicated by an interaction with the measure of linguistic complexity, words per page. *Post-hoc* analyses showed that parents marginally preferred books overall when their target child was in the highest reading ability group (*M* = 3.35) compared to the lowest ability group (*M* = 3.13; *p* = 0.068), with all remaining reading ability groups having intermediate values. But in addition, parents showed a significant negative correlation between their preferences and words per page for only the highest reading group (Pearson's *r* = −0.26, *p* < 0.015). This particular pattern was unexpected and its significance will be discussed in the general discussion.

#### Perceptions of children's preferences

As can be seen in Table 5, parents saw their children's preferences as being governed by factors quite different from their own. None of the measures of cultural prominence significantly predicted children's perceived preferences. Instead, gender—as a main effect and interacting with other factors—was the most prominent element. Overall, there was a main effect of the target child's gender, in that parents of girls provided higher overall perceived preference ratings (Mean perceived preference rating = 3.53) than parents of boys (*M* = 3.45). Although the absolute difference is small, this factor was quite reliable in the model. Parents perceived daughters as liking books in general just a bit more than sons.

The gender of the main character within the book was a significant predictor of children's perceived preferences, but the individual levels only differed from each other in *post-hoc* tests when they were analyzed independently from the other factors. Thus, an ANOVA of children's perceived preferences with the main character's gender as an independent variable was significant [*F*_(2, 84)_ = 4.2, *p* < 0.019] and Tukey's *post-hoc* tests over books showed that books starring female protagonists (*M* = 3.1) were dis-preferred relative to gender neutral books (*M* = 3.4; *p* < 0.04) and also books starring male protagonists (*M* = 3.5; *p* < 0.015) although the latter two categories did not differ from each other. The weakness of this effect appears to stem from a critical interaction with the Target Child's gender: the pattern of dis-preferring books with female protagonists was shown for boys independently (Female protagonist *M* = 2.5 vs. Mixed/Unspecified protagonist *M* = 3.5 vs. Male protagonist *M* = 3.5) but parents of girls reported that their daughters' preferences were not significantly influenced by the gender of the books' protagonists (Female protagonist *M* = 3.5 vs. Mixed/Unspecified protagonist *M* = 3.4 vs. Male protagonist *M* = 3.5). Although the model further showed a significant interaction between the target child's gender and the child experience code, more stringent *post-hoc* tests over the books failed to support it, suggesting that it is smaller in nature.

An additional main effect on the children's perceived preferences was in linguistic complexity: A pearson's correlation confirmed that parents perceived their children as preferring books with fewer words per page (Pearson *r* = –0.43, *n* = 87, *p* < 0.001). In addition, there was a significant interaction between words per page and the target child's reading level. This effect showed a U-shaped pattern as the perceived preference for books with fewer words per page was significant for the two lowest and two highest ability readers (pearson's *r* = −0.27, −0.28, −0.28, −0.36, respectively, all *p*'s < 0.02), but not for children in the middle reading level. Moreover, as can be seen in the correlation co-efficients, the preference for books with fewer words per page was strongest for the best readers. This pattern will be further discussed in the general discussion.

### Summary of experiment 2

The implications of these results will be discussed in the general discussion below, but in summary, the preference study found the following main results: (1) Parents perceived their daughters as liking books more overall than their sons, (2) Parents rated their own preferences as different from those they perceive in their children; (3) Parents preferred books that are more secularly prominent; (4) Parents dispreferred books that star female protagonists and perceived their sons (but not their daughters) as sharing that dispreference; and (5) Parents preferred books with fewer words in them and perceived their children as sharing that preference in general, but both sets of preferences interacted with the reading ability of the child in a non-linear way.

## General discussion

This study conducted two experiments looking at how the features within children's picturebooks relate to each other and how they influence parents' preferences and their perceptions of their children's preferences for those books. Experiment 1 conducted a content analysis on the books themselves and found evidence for different genres of books as well as links between these genres and the gender of the main characters. Experiment 2 found that parents preferences were linked to some (but not all) of the features of the books and their perceptions of their children's preferences interacted strongly with the children's gender.

### Content analysis: coherent classes of books

The content analysis in Experiment 1 found support for a major division of the books which can reasonably be characterized as complexity. Books that were complex along one dimension (more theory of mind, more words per page, more advanced story structure, greater distance from the child's own experience) were likely to be complex along all of the other dimensions. This result is consistent with the results in Schwarz et al. (2015) who similarly found that various dimensions of complexity (linguistic, inferencing, story structure, familiarity of the situations for the child) were predictive of whether experts (speech language pathologists) would classify the book as “easy” or “difficult.” The current results further supported the idea that all the features that contribute to complexity work together coherently across a wide variety of books.

In addition, however, one of these features—the Reflection of the Child's Experience—interacted with the age of the book as well as the gender of the main characters. More recent books were more likely to star non-male characters and were also more likely to closely reflect the child's life. These connections were strong enough that there may be a modern genre of book that is about exploring the regular lives of girls which can be contrasted with a more established genre that is both more magical and more likely to star male characters. It is interesting to speculate about the potential effects—or perhaps, causes—of this modern, girl-oriented genre might be. Barrs (2000) has suggested that among older children, girls have somewhat different reading practices than boys and are more adept at talking about books and being open to emotional engagement with them. Moreover, Millard (1997) has shown that girls are more positive about reading and more motivated to read than boys are (but see McGeown et al., 2012 for evidence that the links are more about gender identification than sex *per-se*). Relatedly, Barrs has suggested that girls' reading practices might be connected to girls' better literacy outcomes relative to boys.

Perhaps early experiences with books that reflect their lives through characters that match their gender helps foster girls' long term positive attitudes toward reading; alternatively perhaps authors and publishers are responding to girl readers by creating books aimed directly at them. Any evaluation of this question would require a more thorough content analysis as well as a historical analysis of picturebook publishing. The current analysis simply coded for the main character's gender and did not include more subtle information about the nature of gender representation and stereotypes within the book. Such information would surely be informative about how girls might be motivated by them. Moreover, as noted previously, this girl-oriented genre may not in fact be modern but instead be ephemeral; that is, books of this type may not be new *per-se* but older examples may simply not be maintained within the canon thus requiring new ones to be created every decade or so.

### Preference analysis

#### Cultural prominence

The preference analysis in Experiment 2 found that parents' own preferences were dramatically swayed by secular factors such as the public acclaim the book had received and how long it has been in print. Acclaim itself was uncorrelated with any of the other content features coded in the books, and the year of publication was linked only to the features that characterized the girl-oriented genre just noted (those books were more recently published). For preferences, however, the more culturally prominent the book was, the more parents preferred it. This result replicates the result from Anderson et al. (2001) that parents prefer more familiar books—parents were likely more familiar with the older books that date from their own childhood as well as books that have received more awards. Indeed, of the 13 books which were dropped for not being familiar to enough parents, none were in the “Classic” age grouping and none had received any awards. Another, related, possibility is that parents are adults, and adults are the people who decide which books win awards and which books are purchased (and therefore, likely to be in print). It is worth noting, however, that parents did not expect their children to be equivalently influenced by secular concerns. It is unclear if this accurately reflects children's distance from secular popularity, or instead reflects the fact that children's access and appreciation of secular concerns may differ from their parents' (and possibly from what was measured in this coding scheme). By the time children are in 2nd grade, their book choices do appear to be influenced by a book's media connections and by the opinions of their peers (Moss and McDonald, 2004; Williams, 2008). Thus, even if parents accurately perceive their pre-school aged children as focusing primarily on the content of the book itself, those children will likely soon be influenced by more external factors.

#### Gender effects

There were a suite of results connecting preferences to gender in various ways. Looking at parents' own preferences, the results showed that parents dis-preferred books starring female protagonists. The cause of this preference is unclear: it was not combined with a preference for books that were distal from the child's experience, so it does not obviously appear to be a dis-preference for the modern, girl-oriented books. However, parents also preferred older books and those books were more likely to star male protagonists; moreover, parents also preferred books with higher critical acclaim and previous work has found that award winning books are very likely to star male protagonists (Gooden and Gooden, 2001). Thus, parents' preferences for books starring male characters may well be a by-product for older and more culturally acclaimed books. Some support for this position comes from the fact that the dis-preference for books starring female protagonists came out most strongly when the character gender factor was analyzed separately from the other factors (including the book's age and level of acclaim). When all those factors are in the same model, it appears that they account for overlapping variability in the preference scores.

Parents' perceptions of their children's preferences were highly influenced by the gender of the child. Specifically, parents perceived their sons (but not their daughters) as dis-preferring books starring female protagonists and to a small extent also books that closely reflected the child's experience. As noted previously, these two content features are themselves correlated with each other, and the gendered preference perceptions further support the idea that there is a distinctive genre of “girl-oriented” books that star female protagonists, and revolve around people, situations and locations familiar to children. Parents perceive their sons as especially disliking these books, and indeed of the eight books that generated the largest discrepancies between girls' and boys' perceived preferences, half were clearly in this genre: *Fancy Nancy, Purplicious, Madeline*, and *My Best Friend is Belle*.

To be fair, there were also several examples of potentially “boy-oriented” books which boys were perceived as liking more than girls. These books included *I Am Sheriff Woody, Dino-Baseball*, and *Airplanes: Soaring! Diving! Turning!*, and these books were also among the eight books generating the largest discrepancies between girls' and boys' perceived preferences—just in the opposite direction. Moreover, there was one book, *Dig*, which was familiar only to parents of boys and so received no perceived preference score for girls at all (it received a score slightly below the average for boys' perceived preferences overall). Interestingly, however, the differential perceived preference scores—for both the “girl-oriented” and the “boy-oriented” books was driven primarily by boys' perceived preferences: boys were perceived as highly preferring the boy-oriented books and highly dis-preferring the girl-oriented books while the girls' perceived preferences scores remained roughly similar. To put this in perspective, only two of the highly discrepant books that favored girls were among girls' top 10 favorites; by contrast, 6 of the 10 most discrepant books favoring boys were among boys' top 10 favorite books.

This asymmetry between parents' perceptions of boys and girls is consistent with previous research showing that while parents, and the culture at large, tend to be accepting of girls engaging in more stereotypically masculine activities (such as playing sports and wearing pants), they tend to be resistant to boys engaging in more stereotypically feminine activities (such as playing with dolls and wearing pink; Kane, 2006; Auster and Mansbach, 2012). The current study did not code the gendered nature of the books beyond simply identifying the gender of the protagonists and no attempt was made to establish whether gender stereotyping (in terms of activities, attitudes, etc.) was linked to the gender of the protagonists. But to the extent that a book simply is more about a girl vs. about a boy, parents' perceptions of their children's preferences is that their daughters like books about both genders while boys do not like books about girls.

More generally, research on the process of children's enculturation into gender roles suggests that parents' perceptions in this domain matter (Maccoby, 2000; Tenenbaum and Leaper, 2002; Martin and Ruble, 2004). Most relevantly, parents perceive girls as being better than boys at the school subject of English (Eccles and Jacobs, 1990), which may be directly related to the results found in Millard (1997) and Barrs (2000) that girls seem to be positively enculturated into the practices of reading and literacy during early childhood much more so than boys are. The results found here suggest that parents see their boys' preferences for books as being contingent on their content to an extent that girls' preferences are not. One consequence of this fact is that girls' were rated as having higher preferences scores overall: girls were perceived as more-or-less liking most books while boys were perceived as only liking some. Thus as children get older, the schema of an open-minded girl reader may increasingly contrast with a picky boy reader who only likes particular books. Whether this accurately comes to describe the children themselves or simply describes the starting point in an enculturation process is beyond the scope of this paper.

#### Family reading practices

The final major finding from these results concerns the power of local family dynamics. Parents' preferences were significantly (though by no means perfectly) correlated with children's perceived preferences, regardless of the child's gender. Given that the overwhelming majority of the parents were female (over 80%) this pattern cannot be attributed to a gender match between the parent and child: sons' and daughters' perceived preferences are both roughly mirrored by their mothers' preferences. As these data are correlational, the directional link between parent and child preferences is impossible to determine. It could be that parents are doing their best to impose their own preferences on their own children. But a more appealing explanation is that that parents enjoy books more when they perceive their children as liking them. Books that lead to a more positive shared book reading experience may be preferred by all the members of the family. That is, the specific local family experiences may be driving this link.

In addition the results showed that parents preferred the books with fewer words per page and perceived their children as sharing that preference. These results potentially point to how parents conceptualize the act of joint book-reading. Previous research has suggested that many of the positive language and literacy outcomes associated with parent-child bookreading may come not from the reading of the text *per-se*, but from the extra-textual conversations that happen around the book (Whitehurst et al., 1994; Sparks and Reese, 2012). Moreover, books with less text in them appear to lead to larger quantities of this extratextual talk (Muhinyi and Hesketh, 2017; Henkaline and Wagner, under review). Thus parents' reported preferences may reflect the fact that they and their chlidren favor books which encourage more extratextual conversations. That is, these families may appreciate the fact that joint book-reading is not just a means to relay a specific story, but is a more wide-ranging cultural act.

Further, the fact that the preference for these less linguistically demanding books is more pronounced for the best readers—both for parents' own preferences and for their children's perceived preferences—suggests that parents may be sensitive to developmental changes. For example, for highly competent readers, linguistically simple books may be favored because the children may be playing a more active role in the actual reading of the book. The average child in this sample was in pre-school and not fully able to read on his or her own; a book where the child is likely to readily succeed in the basic decoding process may lead to more positive parent-child interactions (Baker et al., 2001). Alternatively, the better readers may participate in extratextual conversations in a more enthusiastic or satisfying way: perhaps they are better able to understand and thus discuss the contents of the book than the worse readers. Further research looking at the specific conversations parents and children have while reading the picture books would be needed to determine the nature of the extratextual conversations at different reading levels to determine if there is a shift to discussions about decoding or simply an increase in quantity or quality of the discussions. Regardless, these data suggest that parents are sensitive to the larger cultural uses of joint book-reading.

## Limitations

Perhaps the largest limitation is the correlational nature of the data. As such, these data cannot be used to draw any causal connections, but they do serve as an excellent starting point for creating testable causal predictions. Relatedly, the survey itself focused on breadth as opposed to depth: parents were asked about a large number of books but they were not provided with any opportunities to examine them in detail (only covers were presented) nor were they asked to justify any of their ratings. Thus, the relationships between preference judgments and content features stand on their own without any additional contextual mediation. Further research would do well to interrogate parents more thoroughly about why they preferred some books over others.

Beyond this, the results are limited by the nature of both the books and the participants involved. The set of books that form the core of this study were curated by college-educated women with input from high SES parents and an eye toward the Caldecott awards. Further, the set was settled (and the preference study conducted) in 2011 and so will become dated: to the extent that the content of picturebooks may be changing with time, it will become increasingly important to update this set as well as the preference results that go with it. In addition, the parents who participated in this study were women well connected to social media who had enough interest in children's picturebooks to be willing to complete a 100 question survey about them. They may not constitute a representative sample of the population as a whole. As the results implicated cultural values and family interactions as driving preferences, it is important to be clear about whose values and family types were being examined.

## Conclusion

In our modern society, cultural artifacts like picturebooks are an important part of the child's development context. They provide input to language development, constitute an early rung on the literacy ladder, and are a mechanism of enculturation. These results show how a close examination of the content of these books can help us better understand these artifacts and help us understand how these books are connected to everyday parenting choices.

## Ethics statement

This study was carried out in accordance with the recommendations of The Ohio State University Social and Behavioral Sciences IRB with electronic informed consent from all subjects. All subjects gave informed consent in accordance with the Declaration of Helsinki. Participants were provided with a full consent form online and indicated their consent by checking a box and moving on to the online questionnaire. They were further allowed to end their participation at any time with no penalty.

## Author contributions

The author confirms being the sole writer of this work and approved it for publication. For coding and data collection contributions, please see the Acknowledgments section.

### Conflict of interest statement

The author declares that the research was conducted in the absence of any commercial or financial relationships that could be construed as a potential conflict of interest.

## Footnotes

^1^The division between Regular and Traditional stories was motivated by narratological concerns but many of the coding discrepancies involved drawing the line between these two categories. To insure that effects weren't being driven by a comparatively difficult to parse distinction, all analyses for both studies were thus conducted with the two categories collapsed together, but no qualitative differences were found for any results. The reported analyses all keep them as separate levels.

^2^Note that even with this criterion in place, there were still 27 participants (~18%) in the final sample who reported that their target child was 8 years of age or older. To account for the possibility that even these children were linked to retrospective judgments, all analyses were also conducted with this oldest cohort removed. The results were qualitatively the same, so the 8 – 11 year old target children were retained throughout all analyses.

^3^Inspection of the content codes of the 13 books that had to be dropped suggested that they were quite similar to the rest of the sample with two exceptions: their age and level of acclaim. All of the dropped books were published originally after 1990, and most (77%) were published after 2005; thus, there were no classic books that were completely unfamiliar to the sample of parents who rated the books in Experiment 2. In addition, none of the books had received awards of any kind: by contrast, approximately 40% of the retained sample had received some award of note. For most of the remaining coding categories, the dropped books were very comparable to the retained sample in terms of the extent to which they reflected the child's experiences, the proportion of male & female characters, the average number of words per page, and the level of theory of mind present. The one category where there was a mild difference was in story structure, as books that only depicted a temporal sequence were somewhat under-represented in the dropped sample (8% compared to 28% in the retained sample).

## Appendix

### Picture books mentioned

1 Zany Zoo. (2010). Degman, Lori. Simon and Schuster Books for Young Readers.

Airplanes: Soaring! Diving! Turning! (2008). Hubbell, Patricia. Marshall Cavendish.

Barney and Baby Bop go to School. (1996). Bernthal, Mark S. Lyons Group.

Busiest Firefighters Ever! (1989), Scarry, Richard. Golden Books.

Caps For Sale. (1938). Slobodkina, Esphyr. Harper & Row.

Cat in the Hat, The. (1957). Dr. Seuss. Houghton Mifflin.

Chicka Chicka Boom Boom. (1989). Martin Jr., Bill and Archambault, John. Simon & Schuster Books for Young Readers.

Click, Clack, Moo: Cows that Type. (2000). Cronin, Doreen. Simon & Schuster Books for Young Readers.

Dig. (2004). Zimmerman, Andrea and Clemensha, David. Harcourt.

Dino-Baseball. (2010). Wheeler, Lisa. Carolrhoda Books.

Dumbo. (1955). RH Disney. Golden/Disney.

Eating the Alphabet. (1989). Ehlert, Lois. Harcourt Brace Jovanovich.

Ella the Elegant Elephant. (2004). D'Amico, Carmela and D'Amico, Steve. A. A. Levine Books.

Elmo Loves You. (2005). Albee, Sarah. Dalmation Press, LLC.

Fancy Nancy. (2005). O'Connor, Jane. HarperCollins.

Goodnight Gorilla. (1994). Rathmann, Peggy. Putnam.

Green Eggs and Ham. (1960). Dr. Seuss. Beginner Books.

Happy Birthday Dora! (2010). Michaels, Diana. Simon Spotlight/Nickelodeon.

Harold and the Purple Crayon. (1955). Johnson, Crockett. HarperCollins.

Hello, Goodbye Window, The. (2005). Juster, Norton. Hyperion Book.

Hippos Go Berserk. (1977). Boynton, Sandra. Simon & Schuster Books for Young Readers.

House in the Night, The. (2008). Swanson, Susan Maris. Houghton Mifflin.

I Am Sheriff Woody. (2011). Hashimoto, Meika. Golden Books.

I Just Forgot. (1988). Mayer, Mercer. Golden Books.

I Love Colors. (2009). Miller, Margaret. Little Simon.

JUMP! (2010). Fischer, Scott M. Simon and Schuster Books for Young Readers.

Library Lion. (2006). Knudsen, Michelle. Candlewick.

Lion & The Mouse, The. (2009). Pinkney, Jerry. Litte, Brown Books for Young Readers.

Madeline. (1939). Bemelmans, Ludwig. Viking.

Maisy goes to preschool. (1990). Cousins, Lucy. Candlewick.

Monster at the End of This Book, The. (1971). Stone, Jon. Golden Books.

My Best Friend is Belle. (2006). Marsoli, Lisa Ann. Random House.

No, David!. (1998). Shannon, David. Blue Sky Press.

Olivia. (2000). Falconer, Ian. Atheneum Books for Young Readers.

Polar Express, The. (1985). Van Allsburg, Chris. Houghton Mifflin.

Purplicious. (2007). Kahn, Elizabeth and Kahn, Victoria. HarperCollins.

Shrek!. (1990). Steig, William. Farrar Straus Giroux.

Skeleton Meets the Mummy. (2011). Metzger, Steve. Scholastic Inc..

Tale of Tom Kitten. (1907). Potter, Beatrix. Frederick Warne & Co., Ltd.

Thomas' ABC. (2010). Awdry, Wilbert Vere. Random House.

Thumbelina. (2004). Anderson, Hans Christian and Sneed, Brad. Dial.

Very Hungry Caterpillar, The. (1969). Carle, Eric. Philomel Books.

Where the Wild Things Are. (1963). Sendak, Maurice. Harper & Row.
